# No Evidence of Progressive Proinflammatory Cytokine Storm in Brain-dead Organ Donors—A Time-course Analysis Using Clinical Samples

**DOI:** 10.1097/TP.0000000000004900

**Published:** 2024-01-09

**Authors:** Katarzyna D. Bera, Joel Tabak, Rutger J. Ploeg

**Affiliations:** 1 Nuffield Department of Surgical Sciences, Oxford Transplant Centre, Oxford, United Kingdom.; 2 Oxford University NHS Foundation Trust, Oxford, United Kingdom.; 3 Department of Clinical and Biomedical Sciences, University of Exeter, Exeter, United Kingdom.

## Abstract

**Background.:**

Solid organ transplantation is a cost-effective treatment for end-stage organ failure. Organ donation after brain death is an important source of transplanted organs. Data are limited on the effects of brain injury or donor management on grafts. The consensus view has been that brain death creates a progressively proinflammatory environment. We aimed to investigate time-course changes across a range of cytokines in a donation after brain death cohort of donors who died of intracranial hemorrhage without any other systemic source of inflammation.

**Methods.:**

A donor cohort was defined using the UK Quality in Organ Donation biobank. Serum levels of proteins involved in proinflammatory and brain injury pathways (tumor necrosis factor-alpha, interleukin-6, complement C5a, neuron-specific enolase, and glial fibrillary acidic protein) were measured from admission to organ recovery. Moving median analysis was used to combine donor trajectories and delineate a time-course.

**Results.:**

A cohort of 27 donors with brain death duration between 10 and 30 h was created, with 24 donors contributing to the time-course analysis. We observed no increase in tumor necrosis factor-alpha or interleukin-6 throughout the donor management period. Neuronal injury marker and complement C5a remain high from admission to organ recovery, whereas glial fibrillary acidic protein rises around the confirmation of brain death.

**Conclusions.:**

We found no evidence of a progressive rise of proinflammatory mediators with prolonged duration of brain death, questioning the hypothesis of a progressively proinflammatory environment. Furthermore, the proposed approach allows us to study chronological changes and identify biomarkers or target pathways when logistical or ethical considerations limit sample availability.

## Graphical abstract

**Figure:**
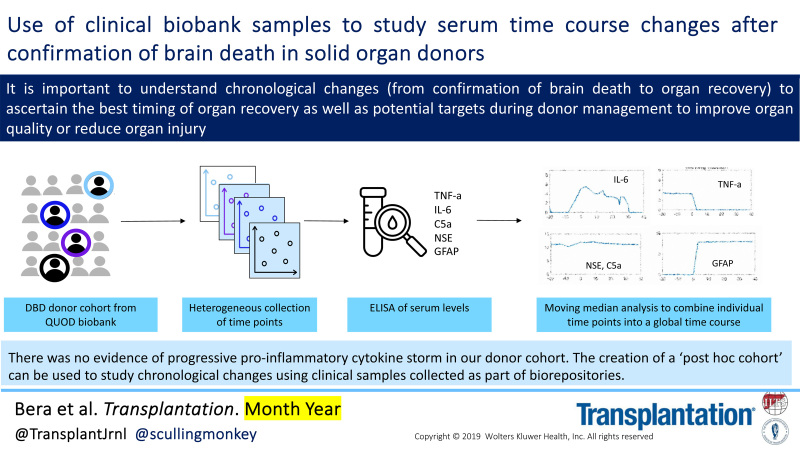

## INTRODUCTION

Organ transplantation is a lifesaving and cost-effective treatment for patients with end-stage organ failure; however, donor organ shortages mean many patients still die while awaiting a transplant. In addition, many retrieved older and higher-risk donor organs are considered untransplantable, declined by transplant centers, and thus not used. Therefore, it is imperative that we improve organ quality and maintain good long-term transplant survival. Donation after brain death (DBD) is the most common source of deceased donor organs worldwide. Although DBD offers a more controlled environment than retrieving organs after circulatory death (when donors will have suffered from a respiratory and cardiac arrest), long-term outcomes between both types of donation remain comparable.^1,2^ Historically, the events surrounding BD have been described as “hostile,” including a catecholamine storm with significant hemodynamic, metabolic, and hormonal changes as well as a progressive release of proinflammatory mediators (eg, tumor necrosis factor [TNF]-alpha and interleukin [IL]-6) and activation of the complement cascade. Chemokine exposure contributes to the long-term trajectory of an organ, leading to fibrosis, impacting long-term function, and ultimately reducing graft survival.^3-5^

To minimize the duration of grafts-to-be in a presumed hostile environment, rapid recovery of organs was adopted, further supported by logistical considerations such as the need for intensive care beds and considerations for donor families. However, retrospective analyses demonstrated that a longer duration of organ donor management in critical care may actually be beneficial for some of the transplanted organs, reducing the rate of delayed graft function (DGF) for renal allografts from younger donors and without a negative impact on transplanted liver or pancreas.^6,7^ The period of brain-dead organ donor management in critical care also offers a therapeutic window of intervention, yet our understanding of inflammatory processes during this period remains limited. Management of the brain-dead donor has evolved and improved in recent history: for example, in the United Kingdom, a “donor care bundle” used by intensivists provides guidance, and donors universally receive corticosteroids,^8^ yet detailed knowledge of how this impacts the pro- and anti-inflammatory balance is currently lacking. Animal models of BD have often been limited to a short time frame (4–6 h).^9-12^ Prior studies of inflammatory serum changes in DBD organ donors measured only a limited number of time points and/or study donors with heterogenous pathologies leading to BD.^13-18^ Understanding details of the time-course surrounding BD is key to developing strategies to reduce organ injury or promote repair, but detailed translation from preclinical studies is lacking and availability of clinical samples is limited.

We hypothesized that the selection of a donor cohort with a shared underlying pathology would enable us to determine the temporal changes of biomarkers of brain injury (neuron-specific enolase [NSE], glial fibrillary acidic protein [GFAP]—to reflect neuronal and glial injury, respectively) and proinflammatory mediators (TNF-α, interleukin-6 [IL-6], and complement), from confirmation of BD through to organ recovery. Understanding the time course can be then used to identify target pathways for treatment even before organ recovery to improve long-term organ quality and graft survival.

## MATERIALS AND METHODS

### Donor Cohort and Serum Sample Selection Process

Serum samples from the UK Quality in Organ Donation (QUOD) biobank were selected to study the BD period between 10 and 30 h (defined as time from confirmation of BD by completion of the second brain stem test through to organ recovery). Inclusion criteria were presence of valid consent, availability of 3 serum samples (1 sample before BD [DB1 sample in QUOD]; 1 sample after confirmation of BD [DB2 sample in QUOD], and 1 sample at the end of donor management period [DB3 sample in QUOD]), intracranial hemorrhage (ICH) as the cause leading to BD, and the documented duration of BD. The following exclusion criteria were used: incomplete set of serum samples, other causes of BD (such as but not limited to hypoxic brain injury, trauma, ischemia, and infection), and documented systemic source of elevated inflammatory markers (trauma, documented infection, eg, pneumonia, urinary infection). Samples were requested from the UK QUOD Biobank, which has collated samples from organ donors across organ retrieval zones in the United Kingdom since 2013. The study includes a cohort of donors who were part of QUOD at the time of application (October 2017). At the point of study cohort design, QUOD had 1271 DBD donors, of which 766 had a documented cause of death as ICH (60.2%); of those, 51 had a full set of samples. Forty-five of 51 donors (88.23%) had a documented BD duration between 10 and 30 h (mean 19.3 ± 7 h).

As the most comparable time point across all donor time courses is the confirmation of BD after the second brain stem test, this was set as *t *= 0 for each donor. Thus, for each patient and each marker, we have a set of 3 sample measurements, 1 before BD (admission sample, DB1), 1 at BD (*t* = 0, DB2), and 1 after BD just before organ recovery (DB3).

To allow the selection of donors who were similar in characteristics other than their individual duration of organ donor management, the 10- to 30-h study duration of BD was broken down into 4 5-h blocks (10–15 h, 15–20 h, 20–25 h, 25–30 h of BD) and for each block (**Figure S1, SDC**, http://links.lww.com/TP/C953). For each group, donors were selected to be balanced for age, gender, body mass index (BMI), and comorbidities (especially hypertension and diabetes); Table 1 summarizes donor characteristics and listing recipient factors known at the point of organ recovery (BMI and age). In addition, all available donors who fulfilled the above-mentioned inclusion criteria but had documented “extremes” of BD duration were also included to allow the study of time course on either side of the core time frame: “short BD” (<10 h) with 2 donors and “long BD” (>30 h) with 5 donors.

**TABLE 1.** Clinical characteristics of study cohort

	Donor							Recipient	
Group	Brain death, h	Age, y	BMI	HTN	DM	Creatinine^a^	Cold ischemia time, h	Age, y	BMI
Group 1 <10 h	5.9 ± 4.0	73.0 ± 8.3	31.7 ± 0.5	2/2	1/2	85.0 ± 8.4	14.1 ± 0.7	67.5 ± 0.7	24.1 ± 0.6
Group 2 10–15 h	13.1 ± 1.9	51.4 ± 11.3	24.8 ± 2.7	0/5	0/5	62.4 ± 9.8	13.6 ± 5.3	51.6 ± 16.5	22.6 ± 5.3^b^
Group 3 15–20 h	17.9 ± 1.4	53.6 ± 6.2	31.7 ± 6.9	3/5	1/5	66.6 ± 18.8	13.2 ± 1.2^c^	55.0 ± 1.8	25.53 ± 5.3^b^
Group 4 20–25 h	22.6 ± 1.9	49.2 ± 8.1	29.0 ± 5.7	1/5	1/5	59.4 ± 24.0	18.3 ± 6.0	47.4 ± 19.3	26.4 ± 5.0^c^
Group 5 25–30 h	26.5 ± 1.6	48.8 ± 10.4	25.9 ± 3.4	1/5	1/5	59.4 ± 24.0	14.4 ± 4.0	42.2 ± 19. 7	27.5 ± 5.5
Group 6 >30 h	33.0 ± 3.6	36.6 ± 15.1	32.7 ± 9.8	1/4^c^	1/5	83.8 ± 33.0	18.2 ± 6.1	40.0 ± 19.8^c^	24.8 ± 1.29^c^
*P* (groups 2–5)	<0.0001	0.833	0.16			0.95	0.70	0.63	0.74
*P*^d^ (groups 1–6)	<0.0001	0.015	0.28			0.45	0.42	0.37	0.72

Overview of created study cohort of 27 donors, group 1 included 2 donors, the remaining groups included 5 donors each. The cohort was created by selecting donors with intracranial hemorrhage, a documented length of brain death and full set of serum samples; donors were matched within each 5 h group of brain death. Extremes of posttransplantation outcome were excluded, as were donors with documented other sources of inflammatory changes such as trauma or infection. Data shown as mean ± SD.

^a^At offer.

^b^n = 2 missing data points.

^c^n = 1 missing data point.

^d^One-way ANOVA.

BMI, body mass index; DM, diabetes; HTN, hypertension.

The study cohort included 27 donors. All included donors had a complete set of serum samples; however, for 6 donors, the exact time point of the first sample collection at the time of admission was not documented; the time and date of admission recorded by NHS Blood and Transplant were used instead. These donors with one missing time point were evenly distributed between the BD groups. The QUOD programme research approval as a Research Tissue Bank (REC ref 13/NW/0017, from North West, Greater Manchester Central Research Ethics Committee) covers the provision of data and research samples for research into improving the quality of organ quality for transplantation.

### Moving Median Analysis

Before the combined analysis of donor serum levels, the Robust regression and Outliner removal method was used to identify outliers. Donors for which at least 4 results were identified as outliers (out of the five measured serum molecules) were excluded. This analysis removed 3 donors—1 from the 25- to 30-h BD duration group and 2 from the >30-h BD duration group, representing the oldest donors in this group, one with a missing admission time point.

Subsequently, data for all remaining donors were combined using moving median plots for each serum level of the molecules. In detail, the time of BD confirmation was defined as *t* = 0 for each donor because this represents the only clinically determined comparable time point between the donors. Thus, for each patient and each marker, we have a set of 3 sample measurements: 1 before BD, 1 at BD (*t* = 0), and 1 after BD. Because the samples before and after BD were obtained at different times for the different patients, the ensemble of measurements across patients contains information about the marker time course before and after BD. We did not directly combine all the measurements into 1 time course for each marker because this would make the strong assumption that markers from different patients follow an identical time course. Here, we make a softer assumption that the time courses from different patients follow qualitatively *similar* trajectories. Following that assumption, we can combine measurements from different patients using a moving median of the time points as long as the window is large enough to account for the variability across patients.

We constructed a moving median for the 20-h period before confirmation of BD and the 40-h period after BD. For the 20-h period before confirmation of BD, we replaced each level by the median of all levels within a 20-h window centered on that point and sampled before *t* = 0. Similarly, for the 40-h period of time after confirmation of BD, we used a 20-h moving window using only levels sampled after *t* = 0. For *t* = 0, the median of all samples collected at this clinical time point was used.

### Enzyme-linked Immune Absorption Assay

**FIGURE 1.**
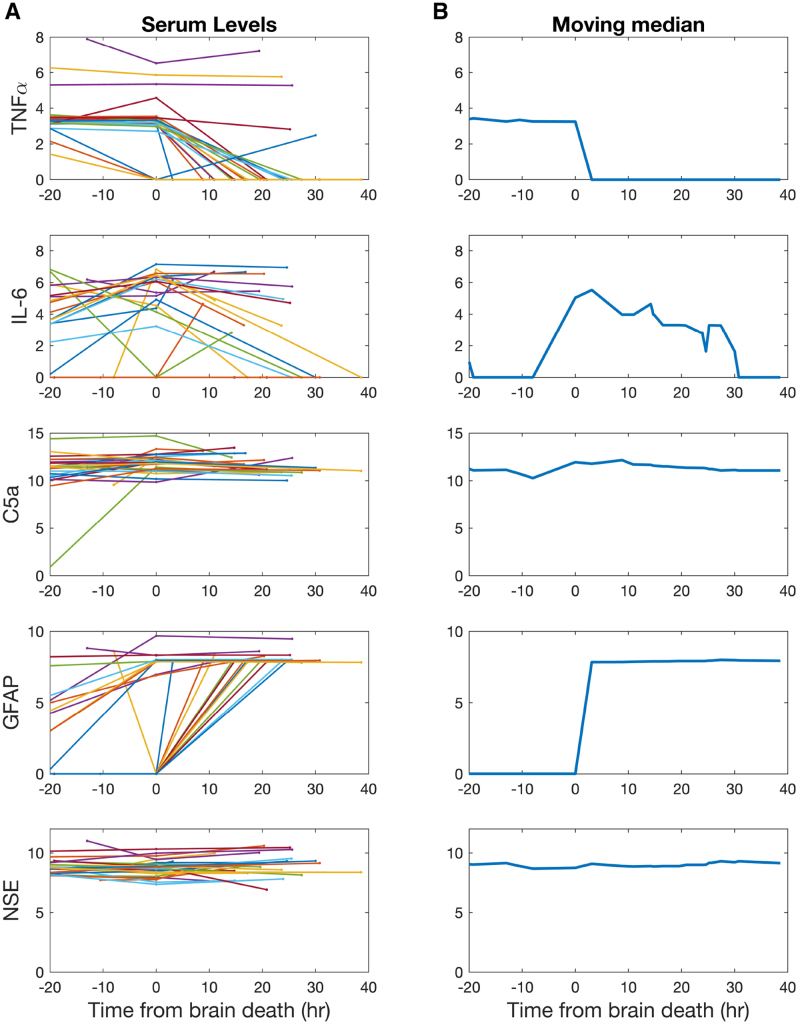
Serum time-course analysis. Each row represents serum levels for 1 of the studied biomarkers: TNF-α, IL-6, complement C5a, GFAP, and NSE, all levels shown in ln(y+1). For each donor, 3 samples based on clinically defined time points (DB1, DB2, and D3) were measured; DB2 was set as *t* = 0 to allow comparison. A and B show analysis of serum levels from individual sample levels to a combined time-course analysis for 24 donors. A, Time courses of serum levels for each donor. Connecting lines depict individual donor trajectories. B, Calculated moving median to define a global time course for each biomarker (moving median window of 20 h). GFAP, glial fibrillary acidic protein; IL-6, interleukin-6; NSE, neuron-specific enolase; TNF-α, tumor necrosis factor-alpha.

All received samples were stored at –80 °C before use. DuoSet Enzyme-Linked Immune Absorption Assay Kits (R&D Systems) for human IL-6, TNF-alpha, complement 5a, NSE, and GFAP were used according to the manufacturer’s instructions. Each sample was measured in duplicates using a Bio-Rad iMark Microplate Reader. MatLab and GraphPad Prism9 were used for data analysis. The log-transformed mean value from the duplicate measurements was used for the time-course plots (Figure 1).

## RESULTs

### Selection of Study Cohort: DBD Donors With Different Durations of BD

The QUOD biobank contains samples from >85% of all deceased organ donors in the United Kingdom, alongside detailed clinical donor and recipient information.^19^ Samples, including serum, are collected at clinically predefined time points: admission (DB1), confirmation of BD (DB2), and end of donor management (DB3). As most DBD organ donors in the United Kingdom have a nonsurvivable ICH, the selection of donors was limited to this pathology. Our selection criteria identified donors with varying durations of BD, as defined by the period from confirmation of BD to the end of organ donor management but before organ recovery. We used donor characteristics collected within QUOD to ensure that the donors across the cohort had a similar profile with regard to age, sex, BMI, and comorbidities. The study was not designed to compare individual organ outcomes and did not include a comparison of groups with known “good” versus “bad” posttransplantation outcomes, such as estimated glomerular filtration rate after 12 mo. This process created a cohort of 24 matched patients with durations of BD ranging from 10 to 30 h and 2 additional groups of all donors with “extreme” durations of BD “short BD” (<10 h) and “long BD” (>30 h).

As outlined in the methods section, available information for all DBD donors within QUOD biobank was used to select a study cohort of donors with similar characteristics but different durations of donor management in intensive care; characteristics of all included donors are displayed in Table 1. One-way ANOVA was used to evaluate the balance between the groups regarding the specified continuous parameters and confirm a balanced of study cohort across all BD groups. When the 2 extremes of short and long BD groups were included, donor age was statistically different between all groups, with notable anticorrelation between donor age and BD duration (*P* = 0.0017, *R*^2^ = 0.33). This might reflect underlying decision making: for example, for younger donors, a prolonged duration of BD might be deemed acceptable, whereas organs from older donors might be only accepted when cold ischemia time and transport duration can be minimized.

### Time-course Analysis

Serum levels of IL-6, TNF-a, C5a, NSE, and GFAP were recorded at time points of admission (sample DB1), after confirmation of BD (sample DB2), and at the end of organ donor management (sample DB3; Figure 1A). As each sample was taken at a clinically defined time point, a comparison was only possible by setting the sample taken after confirmation of BD as *t* = 0. The timeline was divided into the period of ICH management before confirmation of BD (*t* < 0) and a period of organ donor management (*t* > 0) after BD was confirmed. A direct comparison with prior studies of biomarkers of brain injury and proinflammatory mediators is not straightforward because of heterogeneity of underlying pathologies (traumatic brain injury, ischemic or hemorrhagic stroke, and brain injury after cardiac arrest) as well as selection of different time points in a dynamic and evolving situation across studies.^20-28^

Each donor has a unique duration of BD before organs are retrieved, but our work tested the hypothesis that a combination of the data from all donors can be used to define time courses for each biomarker. Looking at all samples (Figure 1A) shows that we cannot simply combine all the time points to define a global trajectory (ie, connecting the dots chronologically). This is because the kinetics of markers that increase and decrease are not identical between donors. To define a global time course for each marker, we instead applied a moving median to the combined set of time points to smooth out the differences between donors. Figure 1B displays the moving median with a window size of 20 h for each of the serum markers from 20 h before *t* = 0 to 40 h after confirmation of BD. The moving median delineates a time course for each measured serum marker over time.

The proinflammatory cytokines IL-6 and TNF-alpha do *not* follow a time course that would indicate a progressively hostile environment. TNF-alpha levels in serum decline from admission to the end of donor management. IL-6 levels show a plateau around confirmation of BD and subsequently decline. NSE (released from damaged neurons) and the complement cascade component C5a both remain elevated from patient admission through confirmation of BD to end of donor management. GFAP, indicating glial cell breakdown, demonstrates a step-like increase after BD and remains constant during the donor management period. Donor BD is believed to occur as a result of irreversible damage to the brain secondary to edema and ultimately brain stem herniation.^29^ Our findings suggest that while neuronal damage leads to a steady level of released NSE from admission onward, there is a step change in glial breakdown products (detected in serum) around the time of confirmation of BD reflective of larger volume glial injury.

## DISCUSSION

Organs from brain-dead donors underperform in the long term and only function at a level comparable with organs obtained from donors after circulatory death^1^; DCD kidneys, for example, experience a higher rate of DGF; however—if DGF remains brief—this does not translate into a worse long-term recipient or graft survival.^30,31^ Following confirmation of BD while patients remain in critical care units, there is a logistical lag until organ retrieval; we are therefore offered a window of opportunity to optimize donors further and improve organ function or reduce damage. In 2017–2018 in the United Kingdom, there were on average 21 h 48 min between the discussion with the donor’s family and start of the retrieval of organs.^32^ In the United States, longer donor management times are reported, which is likely a reflection of different geographical and logistical solutions to the retrieval and transportation of donor organs.^33^ Finland, in contrast, has a median time to procurement of 9.8 and 10.5 h for kidney and liver transplantation, respectively.^34,35^ Our study offers a first characterization of the serum changes during the duration of donor management in UK critical care units, reporting alterations in key proinflammatory mediators as well as markers of central nervous system injury that are not usually present in human serum or plasma in the absence of injury.^36^ The time courses of the different biomarkers obtained by combining individual donor trajectories challenge the current consensus of a progressively proinflammatory environment—the so-called cytokine storm—after BD.

This is the first time-course analysis of serum TNF-alpha, IL-6, and C5a in human DBD donors surrounding the donor care up to organ recovery. Serum changes of NSE and GFAP provide insights into intracranial pathology with increasing release of proteins indicating progressive glial damage around confirmation of BD.

Ultimately, the next steps are to build on our findings to determine how to improve organ quality and posttransplant outcomes. Legally and ethically, treatments and interventions to any potential donor aiming to purely improve transplantation outcomes (beyond measures necessary to stabilize the donor) should be administered after BD is confirmed and consent/authorization given. Logistical, cultural, religious, and ethical considerations need to be considered when the timing and duration of any treatment or intervention are proposed. The future study design will undoubtedly benefit from including patient and public involvement groups with representatives from deceased donor family networks and those on the organ donor register. Therefore, understanding which pathways are amenable to intervention during this time frame is paramount. Targeting pathways or molecules that are already low or declining—whether due to the treatments provided in critical care or as part of a response to the initial injury—would likely not translate into meaningful results. Our work suggests that IL-6 or complement are elevated during donor management and could thus be considered feasible targets for intervention during donor management. Our work is in line with recent animal work that observed a plateau of serum IL-6 and a decline of TNF-alpha after intracranial pressure is experimentally increased.^37,38^ Apart from inhibiting proinflammatory mediators, induction of anti-inflammatory pathways might offer alternative avenues: histological studies of donated DBD kidneys report expression of protective, anti-inflammatory heat shock proteins (heat shock protein 7070 and heme oxygenase 1) alongside known proinflammatory mediators and anti-inflammatory upregulation translating into protecting or restoring renal function.^39^ Both strategies could lead to reduction or elimination of long-term fibrotic changes known to be linked to, for example, macrophage polarization and complement-mediated renal inflammation.^5,18^

Importantly, our study did not provide evidence that supports the previously upheld cytokine storm theory that underpins the perceived need to retrieve organs as quickly as possible. This is in line with some recent cohort analyses, which now propose that a possible early “catecholaminergic storm” might be followed by “storm cooling.” A United Network for Organ Sharing study of cardiac transplantation showed that longer (>42 h) BD times did not result in worse outcomes.^40^ This is in line with a study from Israel that found no correlation between duration of BD and adverse outcomes in cardiac transplantation, although their definition of short BD was <97 h.^41^ Finally, outcomes for liver transplantation after longer duration of BD were associated with better graft survival and fewer short-term complications.^35^

Our work has several strengths, such as the use of high-quality clinical samples from a UK biobank with a high consent rate (>85% of all DBD donors^42^), thus a very good representation of the overall organ donor population. Careful matching of donors and exclusion of those where other factors could affect inflammatory markers (such as due to trauma, anoxia, or infection) allowed us to infer the time course in the “most typical DBD donor” with an isolated ICH as cause for BD. This is in contrast with previous studies that used different inclusion criteria and combinations of pathologies leading to BD that might have different physiological and immunological responses after confirmation of BD and during donor management.^13-18^ The samples that form QUOD biobank are collected on the basis of clinical events, such as confirmation of BD after brain stem testing, rather than set time points; this is of benefit in a situation where we do not yet understand the underlying time course.

The study also has limitations. The final study cohort that allowed for matched characteristics was small (n = 24 donors), which likely reflects the heterogeneity of organ donors managed in critical care across the United Kingdom. This was due to the limited availability of an admission (DB1) sample, which is only present in around 10% of all QUOD DBD samples. Our study design excluded patients where pathology preceding BD would have been likely to produce systemic or neuroinflammation (eg, traumatic brain injury) or are likely to differ in pathology (eg, hypoxia), as these could confound the results. Importantly, animal work suggests that critical care management itself can contribute to systemic inflammatory changes.^37^ It is likely that brain death–related molecular changes follow different trajectories when BD occurs as a consequence of global hypoxia rather than ICH. Finally, it should be noted that our goal was to understand changes representing serum levels of proinflammatory markers present in the donor and thus impacting the transplants-to-be, rather than detailed kinetic analysis including release or breakdown of molecules.

Our work translates more widely by proposing a novel approach for using human samples (including those collected, eg, as part of a biobank), which can be used to study time courses and guide the identification of treatment targets and timings. By creating a “post hoc cohort,” our approach allowed us to use limited clinical samples to study the time courses of biomarkers leading up to organ donation. The development of new therapies typically relies on preclinical animal models. However, in cases where the underlying biology is poorly understood, human pathophysiology differs, or no appropriate models exist, alternative methods are required. Our approach allows us to study serum changes surrounding key events using human samples and can be translated to other clinical settings where repeat sampling is difficult, impossible, or unethical.

## ACKNOWLEDGMENTS

The authors thank Dr Sergei Maslau for his help with the initial selection of the study cohort and Dr Michael Craig for his thoughts and comments on earlier versions of the article. They also thank Dr Meng Sun for his help with accessing further information about the data parameters of the biobank. They thank all organ donors and their families—none of this work would be possible without them. This study was made possible by obtaining samples and data from the UK QUOD Biobank, which is a partnership program between UK academic transplant centers and NHS Blood and Transplant.

## Supplementary Material


