# Supplementary figures and images for: No Evidence of Progressive Proinflammatory Cytokine Storm in Brain-dead Organ Donors—A Time-course Analysis Using Clinical Samples
Source: Transplantation. 2024 Jan 9;108(4):923–9. doi: 10.1097/TP.0000000000004900 (PMC10962432; doi:10.1097/TP.0000000000004900)

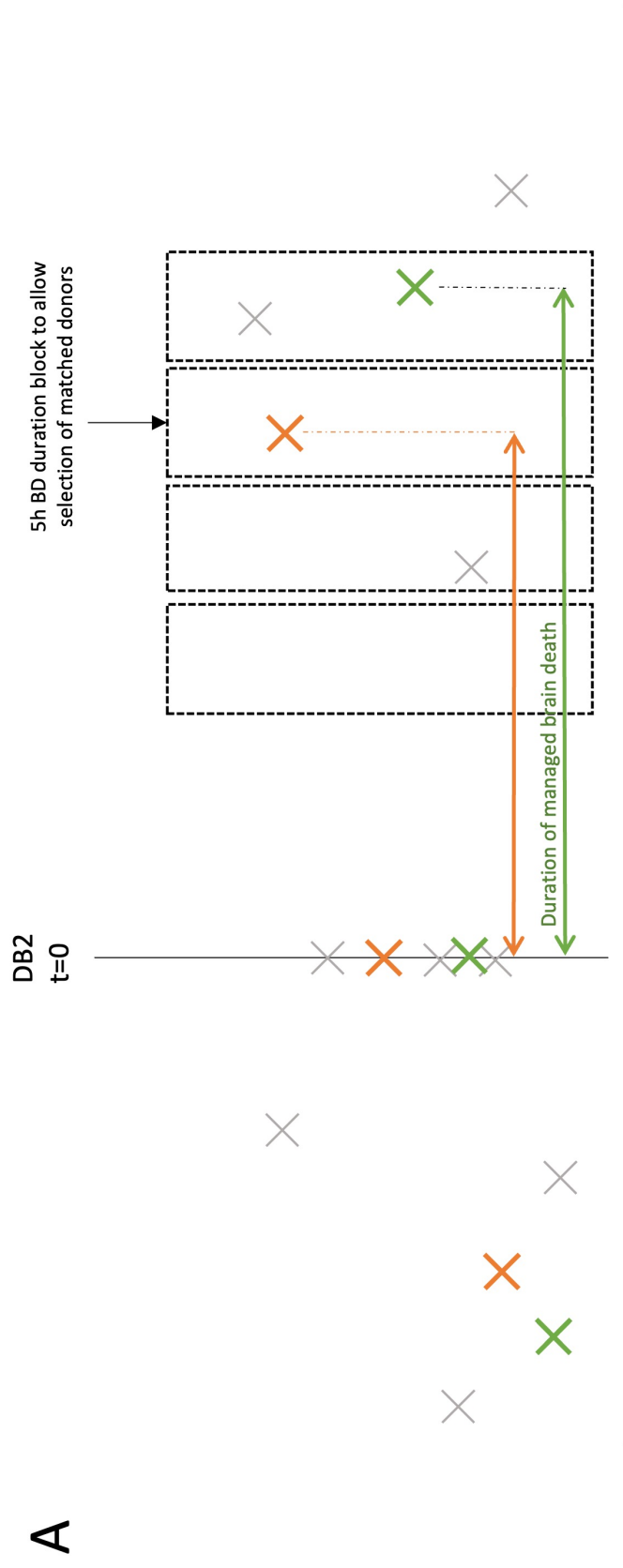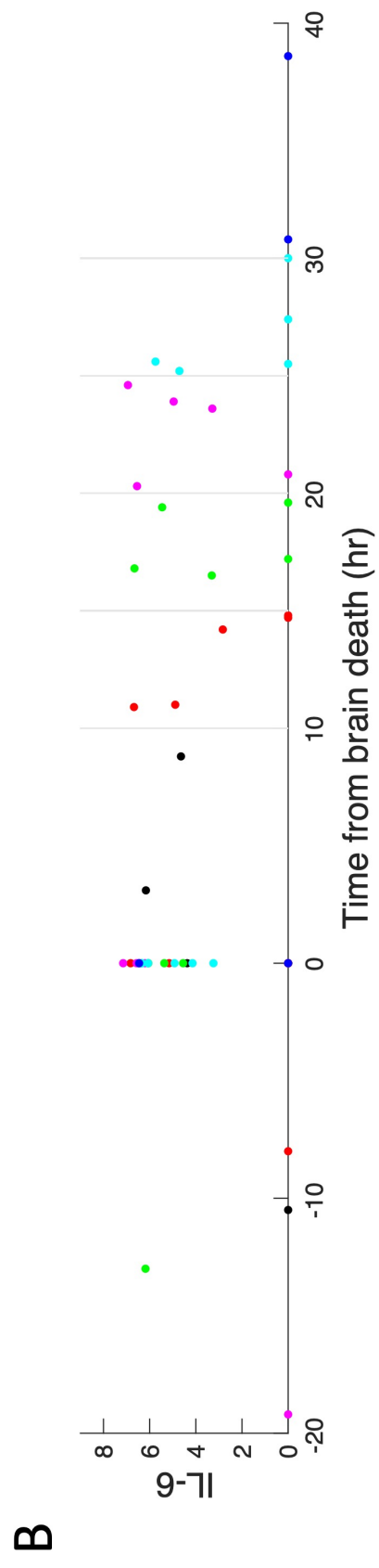

Supplement: Supplementary file 1 [file tpa-108-923-s001.pdf]
